# Systematic review and meta-analysis of cardiovascular event incidence and risk factors in pediatric dialysis patients

**DOI:** 10.1097/MD.0000000000044545

**Published:** 2025-09-19

**Authors:** Xiaoying Zheng, Yingying Ren, Deyue Li, Xueli Lv, Dongmei Wang

**Affiliations:** a Department of Nursing, The First Affiliated Hospital of Heilongjiang University of Chinese Medicine, Harbin, Heilongjiang Province, China; b School of Nursing, Graduate School, Heilongjiang University of Chinese Medicine, Harbin, Heilongjiang Province, China.

**Keywords:** adolescents, cardiovascular disease, children, dialysis, left ventricular hypertrophy, meta-analysis

## Abstract

**Background:**

Cardiovascular disease (CVD) and left ventricular hypertrophy (LVH) are prevalent complications in pediatric and adolescent dialysis patients, elevating morbidity and mortality risks. Despite existing studies on cardiovascular risks, a systematic synthesis of their prevalence and contributing factors is lacking. This study aims to establish an evidence-based foundation to guide clinical interventions.

**Methods:**

We systematically searched PubMed, Embase, Cochrane Library, and Web of Science until March 2025. Cardiovascular events and determinants were descriptively analyzed, while LVH prevalence underwent meta-analysis using a random-effects model. Heterogeneity sources were explored via sensitivity and subgroup analyses (stratified by dialysis modality and study quality), with intergroup differences assessed by mixed-effects meta-regression. Study quality was evaluated using the Newcastle–Ottawa scale (NOS) for observational studies and the Agency for Healthcare Research and Quality checklist for cross-sectional studies. Heterogeneity was quantified with Cochran *Q* and *I*^2^ statistics.

**Results:**

Ten observational studies (5 cohorts, 5 cross-sectional) enrolling 6012 pediatric and adolescent dialysis patients (publication years 1996–2023) were included in the final analysis. The random-effects meta-analysis revealed a pooled LVH prevalence of 56% (95% confidence intervals (CI): 44–69%; *I*^2^ = 81.9%, *P* < .001), indicating substantial heterogeneity. Subgroup analyses demonstrated a numerically higher LVH prevalence in hemodialysis (HD) (66.0%, 95% CI: 52–78%) versus peritoneal dialysis (PD) patients (51.5%, 95% CI: 38–65%), though this difference lacked statistical significance (*P* = .204). Age, gender, and HD modality were independent risk factors. The incidence of cardiovascular-related events was significantly higher in female patients than in males.

**Conclusion:**

Pediatric dialysis patients show significantly higher risks of cardiovascular events and LVH versus controls. This necessitates regular primary echocardiographic monitoring, blood pressure optimization, and risk stratification. Future multicenter studies should: provide optimal dialysis modalities; conduct high-quality research to inform clinical interventions.

## 1. Introduction

Maintenance hemodialysis (HD) is the primary renal replacement therapy for patients with end-stage renal disease (ESRD).^[1]^ Epidemiologic data indicate that approximately 500,000 Americans receive maintenance HD for ESRD.^[2]^ Although HD remains the predominant renal replacement therapy, current technology cannot fully mimic the kidney, an organ with complex metabolic functions and endocrine regulatory mechanisms. Importantly, patients on maintenance HD have a significantly increased risk of major adverse cardiovascular events (acute myocardial infarction, heart failure, and sudden cardiac death, among others) compared to the general population, and such cardiovascular complications have become the most significant lethal factor in this patient population.^[3]^

Patients with chronic kidney disease (CKD) undergoing dialysis face an approximately 20-fold higher mortality risk compared to the age-matched general population, with cardiovascular disease (CVD) being the predominant cause. Young adults who developed advanced CKD during childhood exhibit markedly elevated CVD mortality rates, exceeding those of their age-matched peers by more than 100-fold.^[4]^ The 2016 U.S. renal data system (USRDS) reported that 87% of adults aged ≥45 years had preexisting CVD at ESRD diagnosis, with CVD accounting for approximately 50% of subsequent deaths. In pediatric ESRD patients, CVD-related deaths occurred in 23% of U.S. cases and reached 50% in certain other countries.^[5,6]^

A major adverse cardiovascular consequence of CKD is left ventricular hypertrophy (LVH), which is associated with cardiovascular morbidity and mortality.^[7]^ Children undergoing chronic HD frequently present with LVH and/or diastolic dysfunction, often accompanied by other ventricular pathological abnormalities.^[8]^ In pediatric patients, LVH can be detected in the early stages of CKD and progresses with age.^[9]^ LVH represents a major cause of cardiovascular morbidity and mortality in patients initiating dialysis during childhood.^[10]^ According to studies, up to 66% of maintenance HD patients have LVH, which is a risk factor for cardiovascular events and sudden cardiac death on its own.^[11]^ Furthermore, existing studies demonstrate a paucity of reliable biomarkers for major adverse cardiovascular events in maintenance HD patients, necessitating improved predictive accuracy.^[12]^ Therefore, this study focused on LVH as the primary biomarker for adverse cardiovascular events. We concurrently investigated the prevalence and influencing factors of cardiovascular-related events and LVH, while excluding other biomarkers to maintain analytical focus.

The incidence of cardiovascular-related incidents in children and adolescents receiving dialysis has not yet been evaluated by a systematic review or meta-analysis. The present study provides comprehensive data on the incidence and risk factors of cardiovascular events in pediatric dialysis patients to guide the development of early interventions. Thus, this review aims to systematically analyze published studies regarding the prevalence of cardiovascular-related events and LVH in pediatric dialysis patients through qualitative and quantitative methods.

## 2. Information and methods

### 2.1. Study design and research methodology

This systematic review and meta-analysis were designed, implemented, and reported following PRISMA guidelines. Systematic search, literature screening (performed independently by 2 investigators), quality assessment, data extraction, and statistical analysis were conducted following a predefined study protocol. The protocol was registered on PROSPERO (CRD420251065118). This study is a systematic review and meta-analysis of previously published literature. As no original human or animal data were collected, ethical committee approval was not required.

### 2.2. Data sources and selection process

Four English-language databases – PubMed, Embase, Cochrane Library, and Web of Science – were searched to identify relevant studies published in English before March 2025. The search employed relevant keywords and Boolean operators. For instance, the PubMed search strategy combined the following terms: (Renal dialysis OR HD OR Extracorporeal dialysis) AND (adolescent OR Youth OR children) AND (CVDs OR Adverse Cardiac Event) AND (Cohort Studies OR Cross-Sectional Studies). Similar adapted search strategies were applied to the Cochrane Library, Embase, and Web of Science databases. Additionally, the reference lists of eligible studies were screened to identify further relevant publications. The Appendix (Supplementary Material 15: Search Strategies, Supplemental Digital Content, https://links.lww.com/MD/Q3) contains the comprehensive search tactics for every database.

### 2.3. Inclusion criteria and selection of studies

#### 2.3.1. Inclusion criteria

1. Observational studies (cohort, cross-sectional);
2. Reporting on pediatric or adolescent dialysis patients (defined as 0–19 years per World Health Organization);
3. Reporting the prevalence of LVH and/or cardiovascular events (e.g., heart failure, myocardial infarction, stroke);
4. Providing raw data on the prevalence of LVH or related indicators.

#### 2.3.2. Exclusion criteria

1. Studies that reported only one cardiovascular outcome indicator (e.g., sudden cardiac death, cardiac calcification, arrhythmia) and did not report LVH or cardiovascular events;
2. Studies where the method of LVH diagnosis was unclear or not recommended (e.g., diagnosis based solely on electrocardiogram);
3. Studies that could not distinguish between data on children and adolescents and mixed data on adults;
4. Articles published in languages other than English;
5. Reviews, case reports, conference papers, books, editorials, and letters.

### 2.4. Data extraction and quality assessment methods

All articles reporting on LVH and the incidence of cardiovascular events were initially identified and collected independently by 2 reviewers. Disagreements, if any, were resolved through discussion or consultation with a third reviewer. Then, from the included studies, the same 2 reviewers retrieved the following pertinent information: the name of the first author, the year of publication, the nation in which the study was done, the sample size, the outcome indicators evaluated, the number of positive cases, and the associated prevalence rates. Data on participant sex were also extracted.

Two reviewers independently evaluated the included studies’ methodological quality. Cohort research was assessed using the Newcastle–Ottawa Scale (NOS).^[13]^ The NOS assesses cohort studies based on 8 items across 3 domains: Selection of study groups (representativeness of the exposed cohort, selection of the non-exposed cohort, ascertainment of exposure, demonstration that the outcome of interest was not present at start of study); Comparability of cohorts (based on design or analysis); and Assessment of outcome (adequacy of follow-up, adequacy of follow-up completeness). A maximum of 2 stars can be awarded for comparability and 1 star for each other item, resulting in a maximum score of 9 stars. Low, moderate, and high quality were assigned to studies with scores of 0 to 4, 5 to 6, and 7 to 9 stars, respectively. Quality was evaluated for cross-sectional studies using the standards suggested by the Agency for Healthcare Research and Quality.^[14]^ This tool comprises 11 items, with a maximum score of 11 points. Studies scoring 0 to 3, 4 to 7, and 8 to 11 points were rated as low, moderate, and high quality, respectively.

### 2.5. Data synthesis and analysis

Statistical analyses were performed using Stata software (version 18.0). Data were extracted directly from the original studies or calculated/transformed as necessary. Effect sizes were estimated as the prevalence (or occurrence rate) with corresponding 95% confidence intervals (CI) for each study. Heterogeneity among the included studies was assessed using the *Q*-test alongside the *I*^2^ statistic.^[15]^ *I*^2^ values were interpreted as follows: <25% (low heterogeneity), 25 to 75% (moderate heterogeneity), and >75% (high heterogeneity).^[16]^A fixed-effects model was typically chosen if *I*^2^ < 50% and the *Q*-test *P*-value ≥ 0.1; otherwise, a random-effects model was employed. Sensitivity analyses and subgroup analyses were performed to explore potential sources of heterogeneity. To evaluate publication bias, Egger test was used. Descriptive summaries were used for studies that were not able to be included in the meta-analysis (such as single studies or studies with incompatible data types). At *P* < .05, statistical significance was established.

## 3. Results

### 3.1. Results of literature screening process

**Figure 1.**
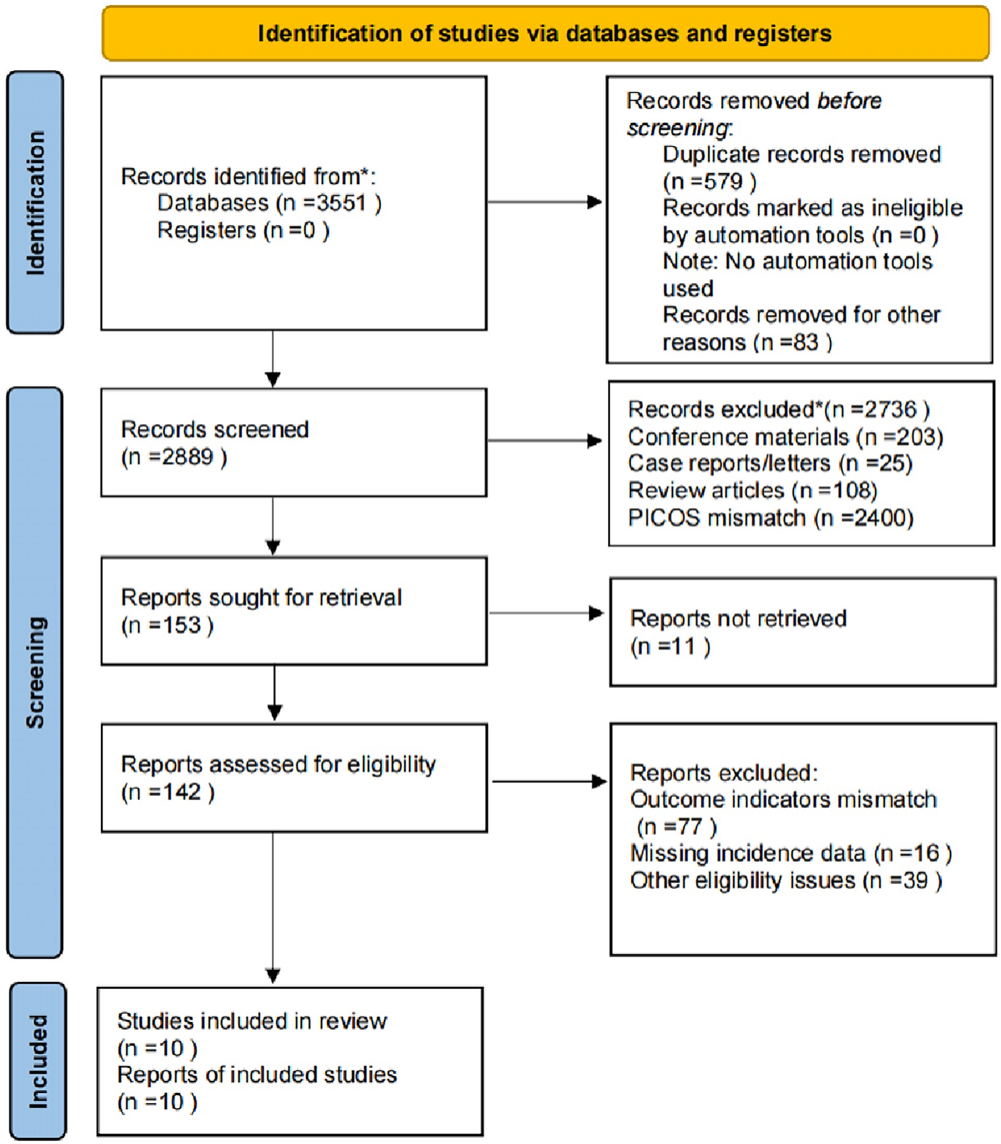
PRISMA flowchart.

By searching 4 English databases, 3551 relevant studies were identified, and 579 duplicates were removed. By reading the full article, 10 studies^[17–26]^ met the inclusion criteria and were included. Four of these studies^[17–20]^ reported the incidence of cardiovascular-related events, which were analyzed descriptively due to the small number of related studies and the large heterogeneity due to issues such as study areas that prevented meta-analysis. The remaining 6 studies^[21–26]^ reported the incidence of LVH in pediatric and adolescent dialysis patients. These studies were subsequently conducted. Figure 1 shows the comprehensive study selection procedure.

### 3.2. Basic characteristics of the included literature

This systematic review included a total of 10 publications (publication years: 1996–2023), covering 6012 pediatric and adolescent dialysis patients. The review aimed to analyze the prevalence of cardiovascular events and LVH. The findings are presented below according to these 2 outcome categories:

Four cohort studies (conducted in the United States, Scotland, Taiwan, and Europe)^[17–20]^ reported on the incidence of cardiovascular events and associated risk factors. These studies had a combined sample size of 5338 participants (range: 381–3097). Cardiovascular event rates varied substantially, ranging from 38.46%^[19]^ to 1.87 per 100 person-years.^[18]^ The main types of cardiovascular events included arrhythmia, cardiac arrest, and valvular heart disease. Core influencing factors identified were age, sex, dialysis modality, and others. Details of the basic characteristics of the included studies are presented in Table 1.

**Table 1:** Basic Characteristics of the 4 literatures with cardiovascular events as an outcome.

Research (yr)	Area	Type of study	Research target	Sample size (male/female)	Number of cardiovascular events	Age range (yr)	Disease rate	Outcome indicator	Factor
Chavers^[17]^ (2002)	America	Cohort study	Pediatric end-stage renal disease patients receiving dialysis	1454 (55%/45%)	452	0–19	31.10%	ICD-9-CM (International Classification of Diseases, 9th Revision, Clinical Modification codes)	(1)(2)(3)(5)
Galiyeva^[18]^ (2019)	Scotland	Cohort study	Pediatric end-stage renal disease patients receiving dialysis	335	–	<18	HD: 1.87/100 person-years; PD: 1.08/100 person-years	Composite outcome of lethal CVD and nonlethal CVD	(1)(2)(6)(7)
Li^[19]^ (2023)	Taiwanese	Cohort study	Children and adolescents on hemodialysis	406 (220/186)	–	0–20	children’s group: 38.46%, youth group: 34.39%	International Classification of Diseases, Ninth or Tenth Revision (ICD9/10) for hospital discharge codes defined in USRDS	(1)(2)(4)(8)
Chesnaye^[20]^ (2016)	European	Cohort study	Pediatric end-stage renal disease patients receiving dialysis	6473 (3634/2839)	–	<19	–	All-cause mortality during dialysis (number of deaths due to cardiovascular events)	(1)(5)(9)

Fatal CVD events: Determined by ICD code in the death record (National Records of Scotland, NRS), defined as death due to any circulatory disease; Non-fatal CVD events: Determined by the first CVD event in the hospitalization record (Scottish Morbidity Records, SMR01), using the same ICD codes as for fatal events (excluding “other circulatory disease”). (1): age; (2): gender-based; (3): racist; (4): dialysis duration; (5): time trend; (6): dialysis modality; (7): primary kidney disease; (8): complication (medicine); (9): Nephrology Specialty Treatment Hours.

CVD = cardiovascular disease, HD = hemodialysis, PD = peritoneal dialysis.

Six studies^[21–26]^ (five cross-sectional and one cohort) reported on the prevalence of LVH, with a total sample size of 674 participants (range: 10–507). The prevalence of LVH ranged from 30% to 75%, with the highest rate (75%) reported in a United States study.^[22]^ Core influencing factors identified were hypertension, anemia, dialysis modality, and others. Table 2 provides specifics of the fundamental traits.

**Table 2:** Basic characteristics of the 6 papers with LVH prevalence as an outcome.

Research (yr)	Area	Type of study	Research target	Age range (yr)	Sample size (male/female	Sample size for occurrence of LVH	Prevalence of LVH	Diagnostic criteria for LVH	Factor
Johnstone^[21]^ (1996)	Australia	Cross-sectional studies	Patients on chronic peritoneal dialysis	1.8–17.5	10(4/6)	3	30%	LVM/HT or LVM/HT2.7 values >95th percentile	(1)(2)(3)(4)(16)
Mitsnefes^[22]^ (2000)	Cincinnati, USA	Cross-sectional studies	Children and adolescents on chronic dialysis	1.8–22	64(32/32)	48	75%	LVMI > 95th percentile	(2)(3)(4)(5)
Seeherunvong^[23]^ (2012)	Miami, USA	Cohort study	Children and adolescents on chronic hemodialysis	6–21	26(-)	14	55%	LVMI > 95th percentile (adjusted for height-for-age)	(6)(7)(16)
Katsoufis^[24]^ (2014)	Miami, USA	Cross-sectional studies	Children and adolescents on chronic hemodialysis	≥6(16.7 ± 2.9)	17(7/10)	9	53%	LVMI ≥ 95th percentile (adjusted for height-age and sex)	(8)(9)(10)(16)
Badawy^[25]^ (2020)	Egypt	Cross-sectional studies	Children with end-stage renal disease on long-term regular hemodialysis	5–16	50(24/26)	33	66%	LVMI above the 95th percentile of normal children and adolescents for gender and age (replacing actual age with height-for-age)	(2)(11)(12)(13)
Bakkaloglu^[26]^ (2011)	Global multicenter	Cross-sectional studies	Children receiving chronic peritoneal dialysis	0.25–19	507(54%/46%)	244	48.1%	LVMI above the 95th percentile of normal children and adolescents for gender and height-for-age	(2)(3)(14)(15) (16)

HT = height, LVMI = left ventricular mass index.

(1): degree of renal impairment; (2): dialysis mode;(3): hypertension; (4): anemia; (5): duration of the disease; (6): FGF-23 level; (7): mineral metabolism disorders; (8): ambulatory blood pressure parameters; (9): type of hypertension; (10): circadian rhythm abnormality; (11): hs-CRP level; (12): IL-18 level; (13): dialysis duration; (14): Fluid overload; (15): obesity; (16): other factors.

### 3.3. Quality assessment of included studies

A total of 10 studies were included: 5 cross-sectional studies and 5 cohort studies. Among the cross-sectional studies, 2 scored 8,^[21,24]^ one scored 7,^[26]^ and 2 scored 5.^[22,25]^ Lower scores in these studies were typically attributed to insufficient methodological rigor, such as inadequate control of confounders or lack of transparency in data collection. Among the cohort studies, one scored 8,^[20]^ one scored 7,^[18]^ one scored 6,^[19]^ and 2 scored 5.^[17,23]^ Lower scores in these studies were commonly due to the lack of clear statements regarding adjustment for confounders and poor completeness of follow-up. Specific details of the quality assessment results are provided in Supplementary Material 13 and 14 (Tables S1 and S2, Supplemental Digital Content, https://links.lww.com/MD/Q2).

### 3.4. Descriptive analysis of cardiovascular event rates

#### 3.4.1. Incidence of cardiovascular disease

The 4 studies reported significant differences in the incidence of cardiovascular-related events.

Chavers^[17]^ studied 1454 pediatric dialysis patients in the United States (1991–1996) and found that cardiac-related events occurred in 31.1% of patients. The most frequent of these events were cardiac arrhythmias (19.6%), valvular heart disease (11.7%), and cardiomyopathy (9.6%). Galiyeva^[18]^ investigated 335 pediatric dialysis patients in Scotland (1961–2013) and reported incidences of 1.87 (95% CI: 1.33–2.43) per 100 person-years in the HD group and 1.08 (95% CI: 0.68–1.47) per 100 person-years in the peritoneal dialysis (PD) group. Fatal events occurred in 20 of these cases. The most common type of CVD was cerebrovascular disease (N = 29), followed by heart failure (N = 13), cardiac arrest/arrhythmia (N = 13), and other (N = 12). Li^[19]^ studied 3910 patients in Taiwan (2003–2017) and found that cardiovascular-related events occurred in 31.83% of patients. Children aged 0 to 12 years had the highest risk, particularly within the first 6 months after dialysis initiation (reaching up to 40%). Chesnaye^[20]^analyzed data from 2000 to 2013 across several European countries, including 6473 patients, and reported that cardiovascular events were the leading cause of death (18.3%); of these, cardiac arrest/sudden death was the most common cause (54.4%).

#### 3.4.2. Age differences

Multiple studies consistently identify age as a significant determinant of cardiovascular-related event risk. Chavers^[17]^ reported a significantly higher incidence of arrhythmias and valvular heart disease in adolescents aged 15 to 19 years compared to the younger age group. Li^[19]^ demonstrated that the highest incidence of composite cardiovascular-related events occurred in children aged 0 to 12 years (peaking at 40% at 6 months post-dialysis), followed by adolescents aged 13 to 20 years (30%). Galiyeva^[18]^ reported that patients younger than 2 years of age at the time of initiation of renal replacement therapy faced a higher risk of all-cause mortality.

#### 3.4.3. Gender differences

Chavers^[17]^ and Li^[19]^ consistently reported elevated risks of cardiovascular-related events in female patients. Chavers^[17]^ demonstrated significantly higher prevalences in females versus males for both arrhythmias (23% vs 17%; *P* = .004) and valvular heart disease (14% vs 10%; *P* = .03). Li^[19]^ found the risk of cardiovascular-related events was significantly greater in females aged 13 to 20 years (hazard ratio (HR) = 2.08) compared to males (HR = 0.75; *P* < .01). A study by Galiyeva^[18]^ showed that for male dialysis-treated patients with primary renal disease was associated with a higher rate of cardiovascular-related events.

#### 3.4.4. Racial and dialysis modality differences

Chavers^[17]^ specifically examined racial disparities, revealing significantly higher prevalences in Black versus White patients for arrhythmias (27% vs 16%), valvular heart disease (16% vs 9.5%; *P* = .002), and cardiomyopathy (14% vs 7.5%; *P* = .001). Chesnaye et al’s multinational European study^[20]^ employed adjusted HRs with propensity score-matched (PSM) analysis. The primary analysis demonstrated higher mortality risk in HD versus PD patients (adjusted HR = 1.39, 95% CI: 1.06–1.82; PSM HR = 1.46, 95% CI: 1.06–2.00). Especially in the first year of dialysis (HD/PD adjusted HR = 1.70, 95% CI: 1.22–2.38, PSM HR = 1.79, 95% CI: 1.20–2.66), when the start of dialysis was >5 years of age (HD/PD adjusted HR = 1.58, 95% CI: 1.03–2.43, PSM HR = 1.87, 95% CI: 1.17–2.98), and when the child was seen for only a short period before starting dialysis (HD/PD adjusted HR = 6.55, 95% CI: 2.35–18.28, PSM HR = 2.93,95% CI: 1.04–8.23).

### 3.5. Results of meta-analysis

#### 3.5.1. Results of analysis of the prevalence of LVH in pediatric and adolescent dialysis patients

**Figure 2.**
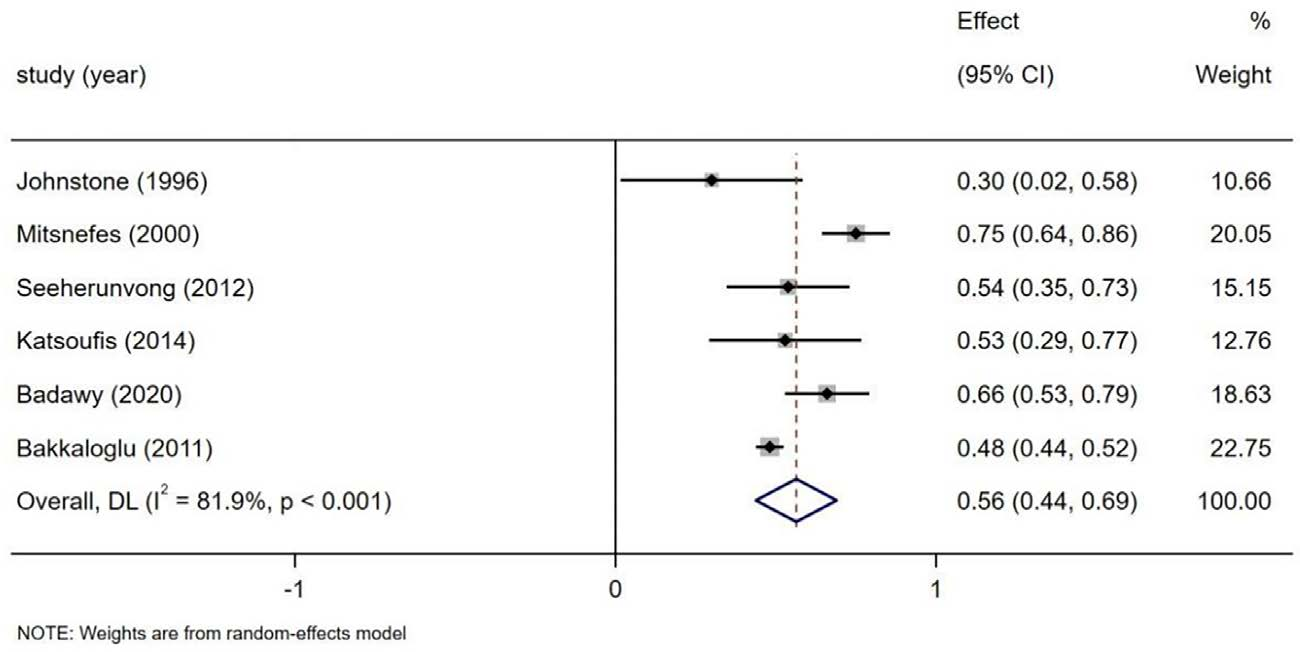
Forest plot of prevalence of LVH. LVH = left ventricular hypertrophy.

The combined prevalence of LVH in pediatric and adolescent dialysis patients was estimated at 56% (95% CI: 44–69). Significant heterogeneity was observed across studies (*I*^2^ = 81.9%, *P* < .001) (Fig. 2).

The prevalence was 51% (95% 34.6–68.3) in the PD group and 66% (95% 51.2–80.7) in the HD group, with no statistically significant difference between groups (*P* = .204). This suggests dialysis modality may not be the primary driver of prevalence differences, although the significantly higher absolute prevalence in the HD group warrants attention. This elevated prevalence in HD patients aligns with Mitsnefes,^[24]^ who reported higher LVH prevalence in the HD cohort compared to the PD group (Table 3). Supplementary Materials 1 and 2 contain forest plots of subgroup analysis (Figs. S1 and S2, Supplemental Digital Content, https://links.lww.com/MD/P1000).

**Table 3:** Results of subgroup analysis.

Subgroup	Number of studies	Disease rate (%)	95% CI	Inter-study heterogeneity		Model selection	Difference between groups (*P*)
				*I*^2^ (%)	*P*		
Dialysis modality							
Peritoneal dialysis	3	51.5	34.6–68.3	76.4	.014	Random effects model	.204
Hemodialysis	4	66.0	51.2–80.7	67.5	.026	Random effects model	
Quality of research							
High-quality research	2	43.5	25.3–61.7	32.3	.224	Fixed–effects model	.056
Medium quality research	4	56.4	43.8–69.0	88.1	.000	Random effects model	

The prevalence of LVH was 43.5% (95% CI: 25.3–61.7) in high-quality studies and 56.4% (95% CI: 43.8–69.0) in moderate-quality studies. The intergroup difference approached statistical significance (*P* = .056), suggesting study quality may significantly influence results, with high-quality studies yielding more conservative prevalence estimates (Table 3). Supplementary Materials 3 and 4 contain forest plots for subgroup analysis (Figs. S3 and S4, Supplemental Digital Content, https://links.lww.com/MD/P1000).

#### 3.5.2. Sensitivity analysis

The influence of individual studies was evaluated using a leave-one-out sensitivity analysis. These analyses demonstrated that the pooled LVH prevalence estimates were robust, with no undue influence from individual studies. After sequential exclusion of individual studies, prevalence estimates ranged from 0.522 (95% CI: 0.425–0.619) to 0.595 (95% CI: 0.463–0.728) (Figs. S5 and S6, Supplemental Digital Content, https://links.lww.com/MD/P1000). These results further substantiate the reliability of these findings.

#### 3.5.3. Publication bias test

**Figure 3.**
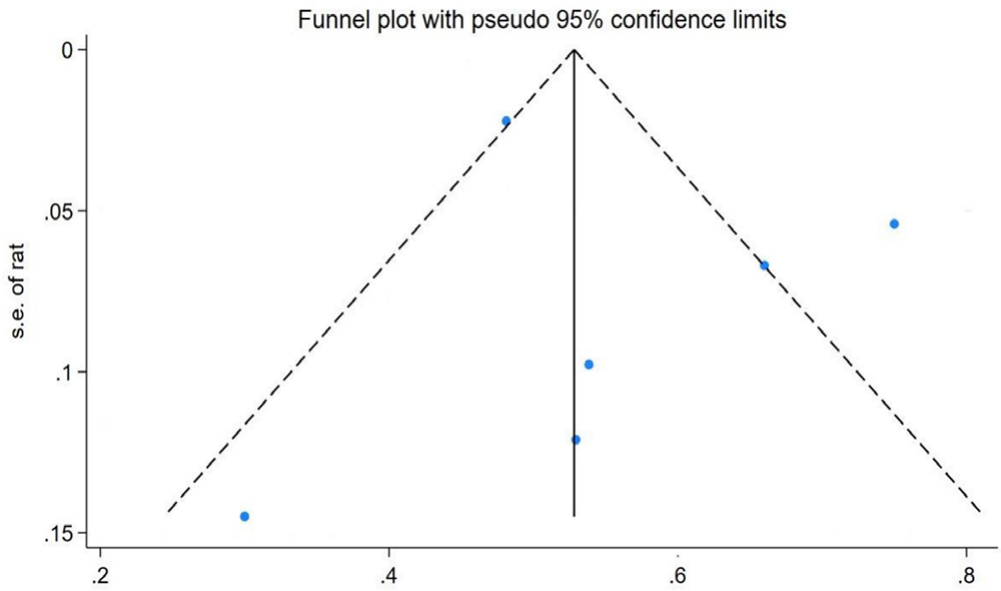
Funnel diagram.

There was little chance of publication bias, according to Egger test (slope coefficient = 1.147, *t* = 0.68, *P* = .534) (Fig. S7, Supplemental Digital Content, https://links.lww.com/MD/P1000). As presented in Figure 3, the funnel plot for LVH prevalence in pediatric and adolescent dialysis patients demonstrates overall symmetry in study point distribution. Although minor asymmetry was observed in smaller studies, statistical testing (*P* > .05) indicated no statistically significant influence of publication bias on overall results. Collectively, statistical testing and visual inspection indicate that meta-analysis conclusions are unlikely to be affected by publication bias.

## 4. Discussion

### 4.1. Key findings

This study is the first to do a systematic review and meta-analysis of the prevalence of LVH and cardiovascular events in juvenile and adolescent dialysis patients. A total of 10 studies were included, spanning multiple regions (the United States, Scotland, Taiwan region (China), and European countries). Analyses demonstrated that the prevalence of cardiovascular events and LVH was influenced by age, sex, dialysis modality, and study quality. Studies predominantly originated from high-income countries (90%, n = 9), with only one from a middle-income nation (Egypt). Most reported prevalence stratified by age, sex, and dialysis modality (HD vs PD), using standardized LVH diagnostic criteria to ensure methodological rigor.

Descriptive analysis of cardiovascular events revealed significant incidence variations, with age, sex, and dialysis modality as key influencing factors. Children ages 0 to 12 and adolescents ages 13 to 20 were at the highest risk, especially during the first 6 months of dialysis. Females exhibited an elevated incidence of arrhythmias and valvular disease, while cardiomyopathy incidence was higher in Black versus White patients.

The pooled LVH prevalence was 56% (95% CI: 44–69), with substantial heterogeneity (*I*^2^=81.9%, *P* < .001). HD patients showed higher absolute prevalence (66.0%; 95% CI: 51.2–80.7) versus PD (51.5%; 95% CI: 34.6–68.3), though non-significant (*P* = .204). Notably, high-quality studies reported lower LVH prevalence (43.5%; 95% CI: 25.3–61.7) than moderate-quality studies (56.4%; 95% CI: 43.8–69.0), a non-significant difference (*P* = .056).

In summary, cardiovascular events and LVH are prevalent, life-threatening complications in this population, with substantially higher prevalence versus the general population. These findings support implementing targeted interventions and enhanced cardiovascular monitoring to reduce morbidity and mortality.

### 4.2. Comparison with existing studies

Multiple studies indicate that over 70% of patients initiating dialysis develop left ventricular (LV) hypertrophy, an independent risk factor for cardiac mortality, with approximately 45% of dialysis patients dying of cardiac causes.^[27]^ Foley’s study of 433 adult ESRD patients demonstrated LVH prevalence of 74% at baseline, significantly associated with advanced age, hypertension, and anemia.^[28]^ We observed similarly high LVH prevalence in pediatric/adolescent dialysis patients (pooled rate: 56%), with hypertension and anemia as key determinants. Foley’s study demonstrated a significant correlation between LVH and elevated mortality. Our results further revealed a higher prevalence of LVH in pediatric dialysis patients compared to adults (reaching 75% in some studies^[22]^), suggesting that the pediatric cardiovascular system appears more vulnerable to the uremic milieu. Additionally, while Foley identified LV systolic dysfunction as an independent predictor of mortality, our study, though not directly analyzing systolic function, found LVH, as its precursor manifestation, similarly associated with poor prognosis.

Groothoff’s investigation (2005) reported cardiovascular mortality in pediatric ESRD patients 30-fold higher than the general population,^[5,29]^ with prolonged dialysis exposure increasing disease risk. Our study corroborates these findings and identifies the highest risk of cardiovascular-related events in younger children (0–12 years) and adolescents (13–20 years), particularly within 6 months following dialysis initiation (incidence up to 40%). Furthermore, whereas Groothoff emphasized prognostic improvement through early transplantation, our subgroup analysis indicated a trend toward higher LVH prevalence in HD patients (66.0%) versus PD (51.5%). Although this difference lacked statistical significance (*P* = .204), it suggests that dialysis modality may influence cardiac remodeling through hemodynamic variations.

We observed a lower LVH prevalence in high-quality studies (43.5%) versus moderate-quality studies (56.4%), aligning with Foley et al.‘s emphasis on methodological rigor.^[5]^ This disparity likely reflects more stringent control of confounding variables in high-quality studies. Additionally, we observed substantial heterogeneity in our meta-analysis (*I*^2^ = 81.9%), potentially attributable to regional variations, divergent diagnostic criteria, or differential follow-up durations. This finding is consistent with the heterogeneity issues in pediatric ESRD studies reported by Groothoff.^[5]^

### 4.3. Strengths and limitations

The prevalence of cardiovascular-related events and LVH in children and adolescent dialysis patients is being investigated for the first time in this study’s systematic review and meta-analysis, addressing a significant gap in this research area. We not only quantified the prevalence of cardiovascular-related events and LVH but also investigated key determinants, including age, gender, and dialysis modality, thereby offering multidimensional evidence for clinical practice. Inclusion of studies extending the upper age limit to 22 years may introduce potential heterogeneity; however, sensitivity analysis demonstrated robust overall findings. All procedures adhered strictly to PRISMA guidelines, employing standardized diagnostic criteria and quality assessment tools to ensure scientific rigor. Key limitations include the predominant representation of high-income countries, with only one study from a middle-income nation (Egypt), potentially limiting generalizability to low-income settings. Furthermore, restrictions to English-language publications possibly excluded relevant studies, compromising comprehensiveness. Although we systematically searched the literature up to March 2025, studies published in March 2024 to 2025 were not included in the analysis because of insufficient follow-up time or inconsistent outcome metrics, which may affect the reflection of the most recent clinical practice. Future studies need to extend the follow-up time and establish a standardized reporting system for cardiovascular complications, especially for the special population of pediatric dialysis patients. While arrhythmias and valvular disease were reported in 4 studies, quantitative synthesis was restricted by methodological variability. Future studies should standardize definitions to facilitate pooled analyses.

### 4.4. Significance of results

The study further demonstrated that the prevalence of cardiovascular-related events and LVH in children and adolescents on dialysis is higher than in the general population. Most studies indicate that hypertension and dialysis modality are key factors influencing the prevalence of these conditions in dialysis patients. This suggests the need to enhance monitoring of cardiovascular function early in the treatment course and to implement targeted interventions. The findings also inform public health policies that seek to reduce the risk of disease by enhancing early disease surveillance and optimizing dialysis modalities.

### 4.5. Discussion

This study shows that the prevalence of cardiovascular-related events and LVH is higher in young dialysis patients and is significantly influenced by factors such as age, gender, and dialysis modality. The high incidence of non-LVH complications – particularly early-onset arrhythmias – underscores the need for routine cardiovascular surveillance beyond echocardiographic LVH assessment in this population. Although the findings were subject to limitations such as geographic variation, the number of included studies, and study quality, they still provide an important basis for early intervention and optimized management of the disease. Further large-sample, multi-center, high-quality studies are needed to improve global evidence-based medical practice and enhance the long-term prognosis among this high-risk population.

## Author contributions

**Conceptualization:** Xiaoying Zheng, Yingying Ren, Deyue Li.

**Data curation:** Xiaoying Zheng, Yingying Ren, Deyue Li, Dongmei Wang.

**Formal analysis:** Xiaoying Zheng.

**Investigation:** Deyue Li.

**Methodology:** Dongmei Wang.

**Resources:** Yingying Ren, Xueli Lv.

**Software:** Deyue Li, Xueli Lv, Dongmei Wang.

**Supervision:** Xiaoying Zheng, Yingying Ren.

**Validation:** Xiaoying Zheng, Dongmei Wang.

**Visualization:** Xueli Lv.

**Writing – original draft:** Xiaoying Zheng, Yingying Ren, Deyue Li, Xueli Lv, Dongmei Wang.

**Writing – review & editing:** Xiaoying Zheng, Yingying Ren, Deyue Li, Xueli Lv.

## Supplementary Material


