# Supplementary material for: Systematic review and meta-analysis of cardiovascular event incidence and risk factors in pediatric dialysis patients
Source: Medicine (Baltimore). 2025 Sep 19;104(38):e44545. doi: 10.1097/MD.0000000000044545 (PMC12459514; doi:10.1097/MD.0000000000044545)
Supplement: Supplementary file 1 [file medi-104-e44545-s001.pdf]

Figure S1 : Subgroup analysis forest map - dialysis approach

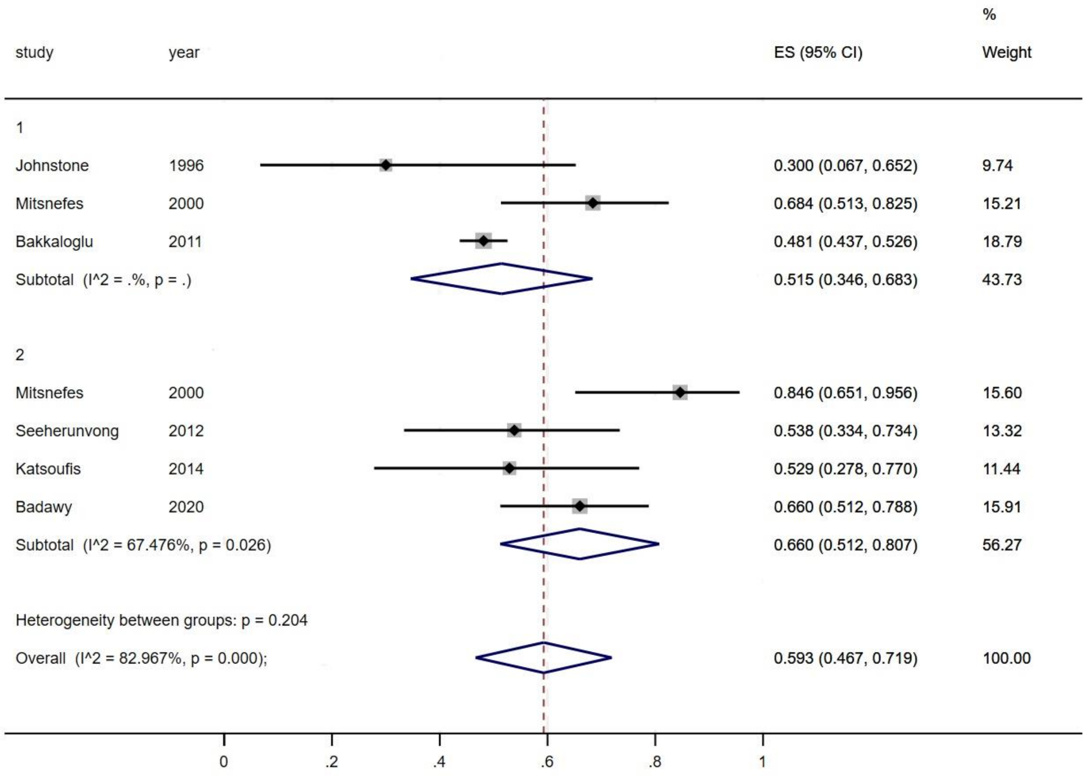

Figure S2: Subgroup analysis forest map - dialysis approach

Subgroup 1 (Group 1) was manually calculated because it contained only 3 studies and heterogeneity was not automatically displayed.This is a supplement to the heterogeneity results not shown in S1.

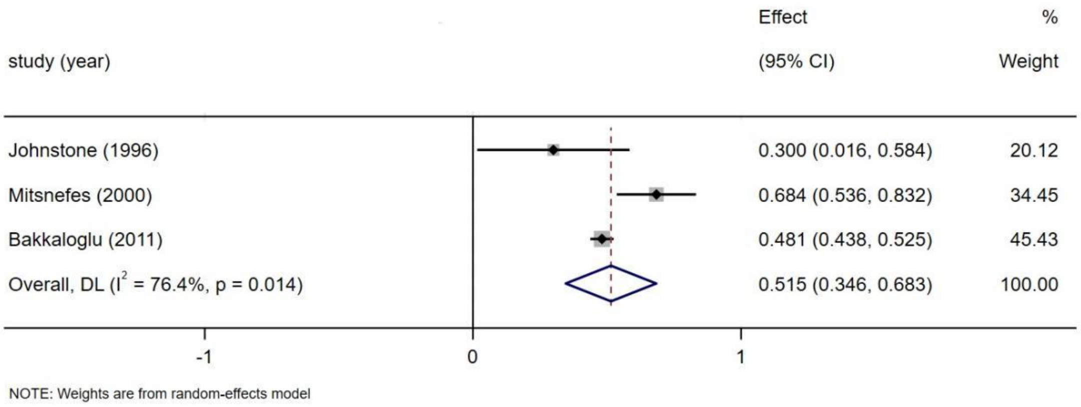

Figure S3: Subgroup analysis forest map - high and low quality of the study

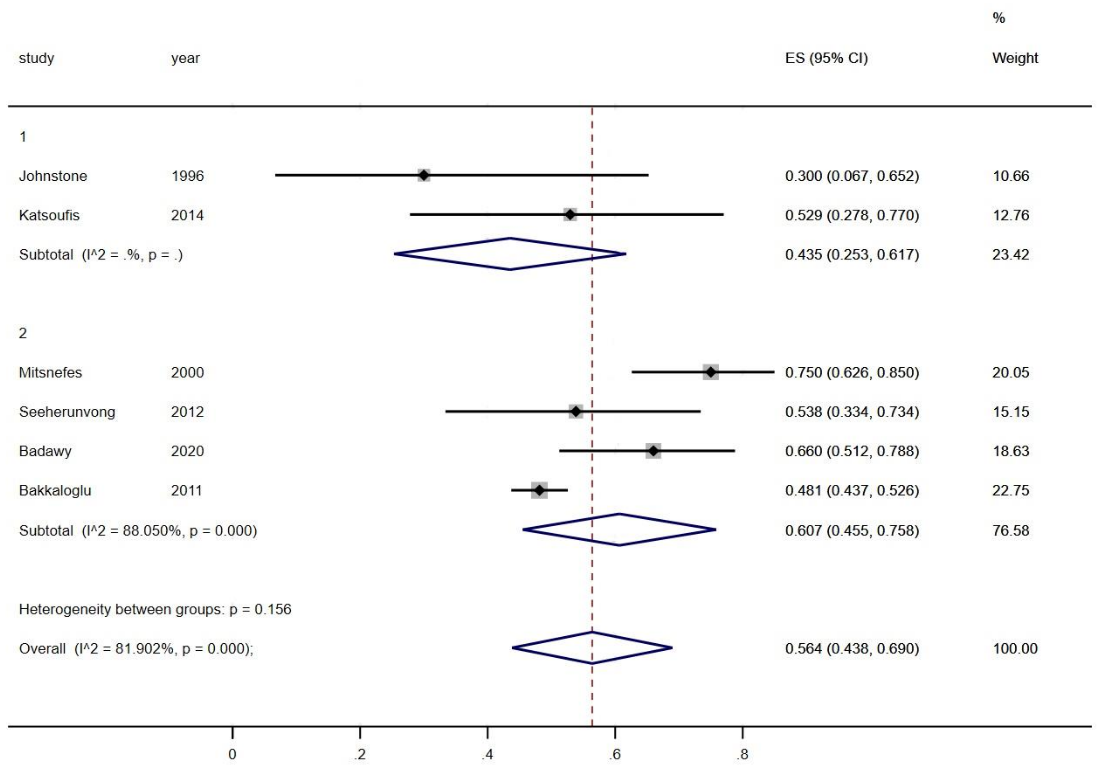

Figure S4: Subgroup analysis forest map - high and low quality of the study

Subgroup 2 (Group 2) was manually calculated because it contained only 2 studies and heterogeneity was not automatically displayed.This is a supplement to the heterogeneity results not shown in S3.

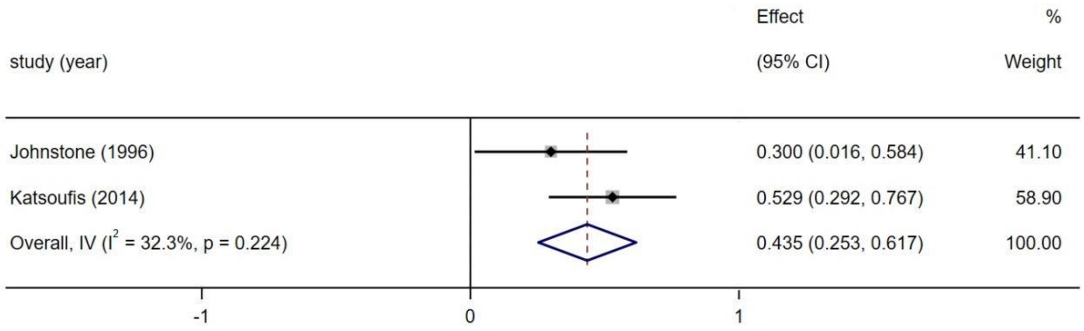

Figure S5: Sensitivity analysis results map

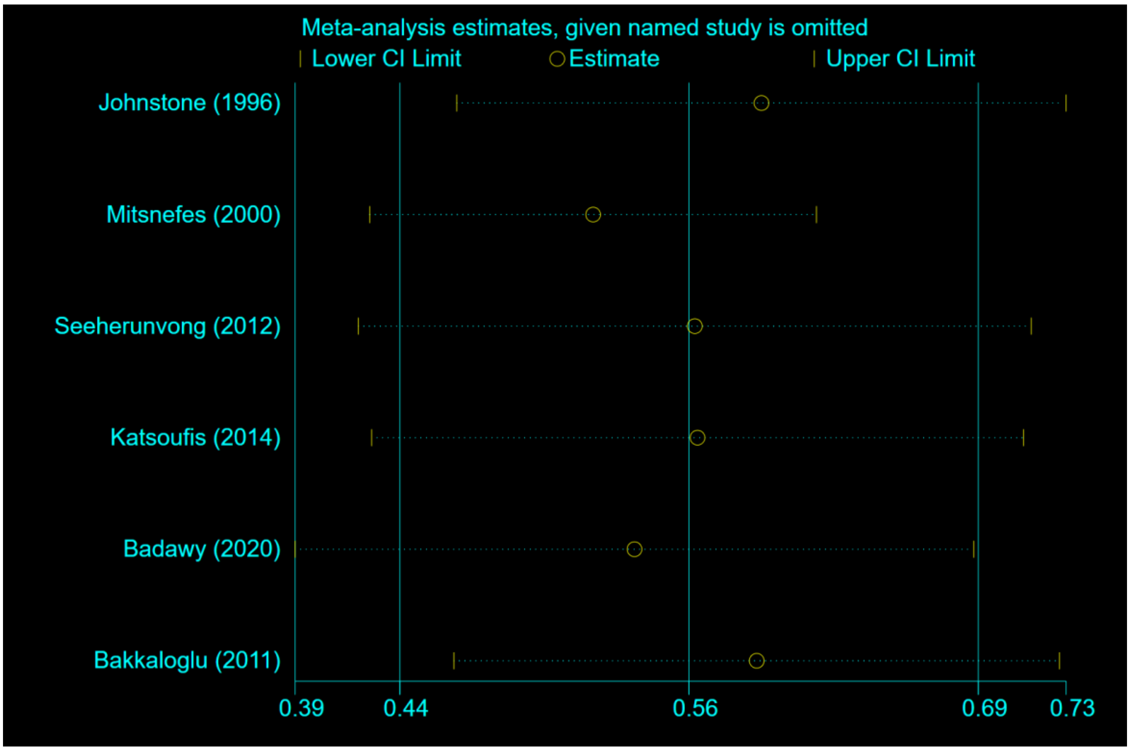

Figure S6: Sensitivity analysis results map

Study omitted	Estimate	[95% Conf.	Interval]
**Johnstone (1996)**	**.59542799**	**.46296698**	**.727889**
**Mitsnefes (2000)**	**.52222157**	**.42513195**	**.61931109**
**Seeherunvong (2012)**	**.56642854**	**.42015222**	**.7127049**
**Katsoufis (2014)**	**.56760538**	**.42592677**	**.70928401**
**Badawy (2020)**	**.54019696**	**.3926596**	**.68773437**
**Bakkaloglu (2011)**	**.5933463**	**.46170938**	**.72498322**
**Combined**	**.56391904**	**.43816726**	**.68967081**

Figure S7: Graph of Egger's test results

Number of studies = **6** Root MSE = **2.489**

Std_Eff	Coefficient	Std. err.	t	P>|t|	[95% conf.	interval]
slope	**.4858894**	**.0780058**	**6.23**	**0.003**	**.2693104**	**.7024683**
bias	**1.147404**	**1.689769**	**0.68**	**0.534**	**-3.544148**	**5.838956**

Test of H0: no small-study effects P = **0.534**

Figure S8: Web of science 14 articles, 14 articles in 2024, 2 articles in May 2025.

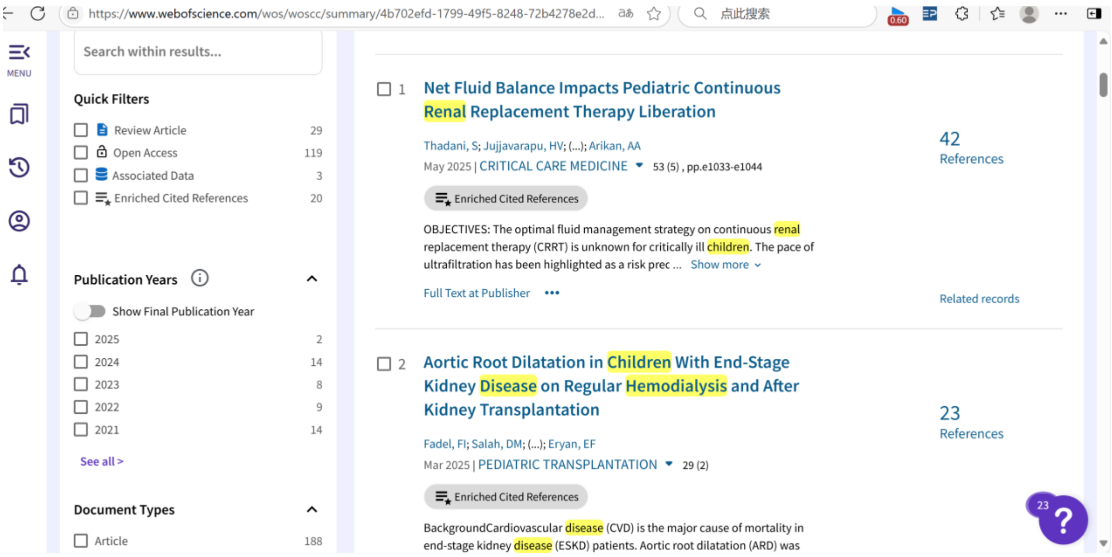

Figure S9: Cochrane: 2 articles, 1 review, 1 trial.

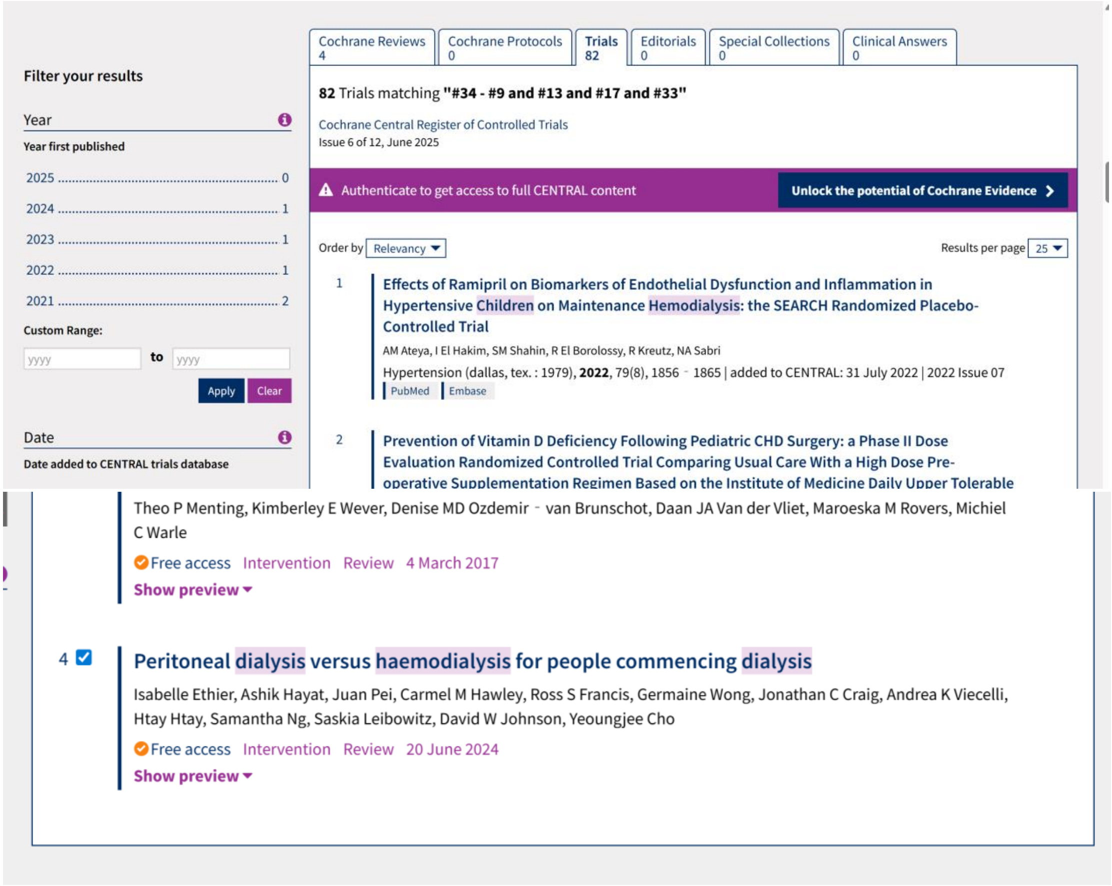

Figure S10: Embase 2024-2025 March 107 articles.

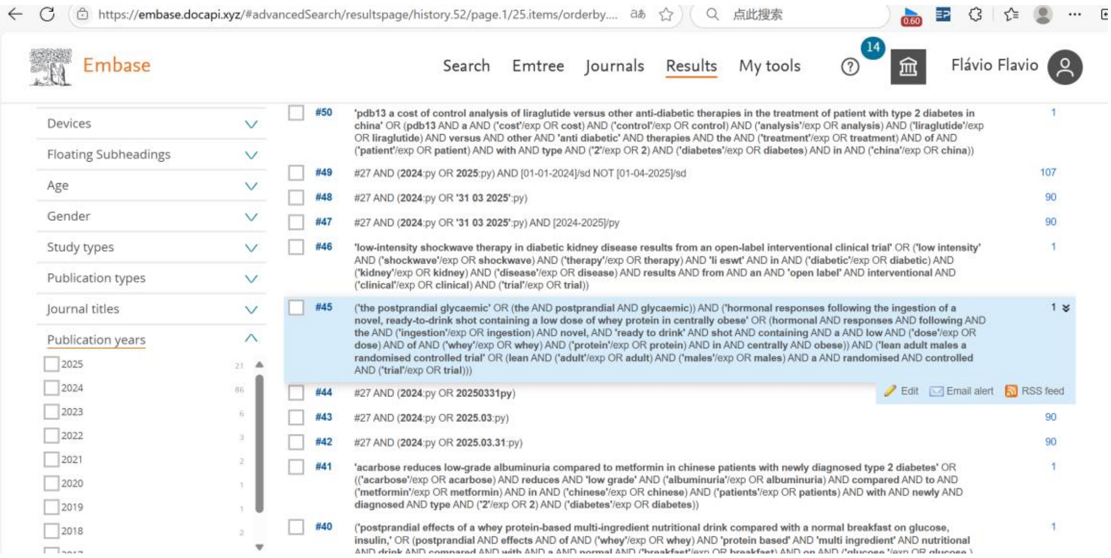

Figure S11: Pubmed 25 articles.

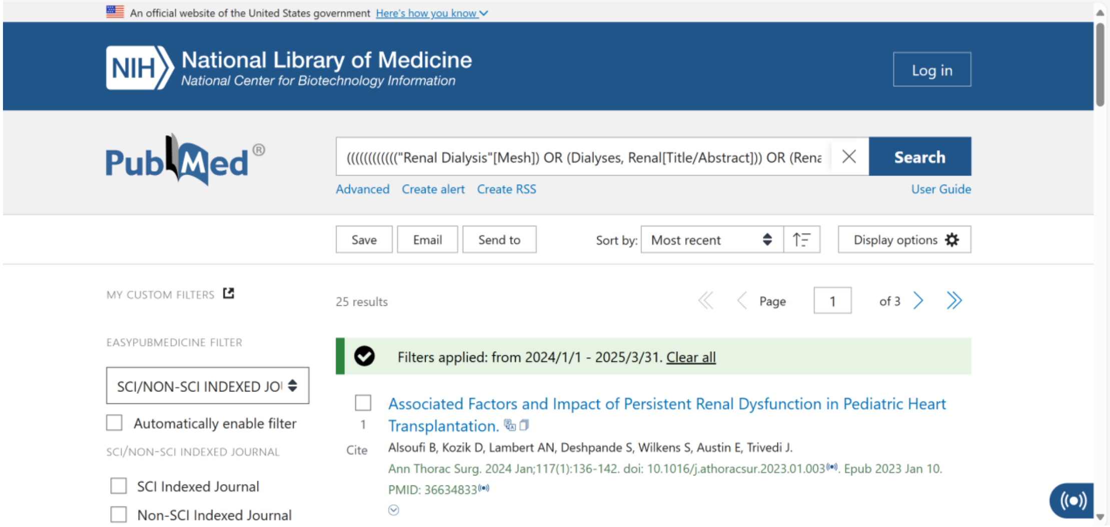

Figure S12:The updated PRISMA flowchart for the 2024 – 2025.3 search

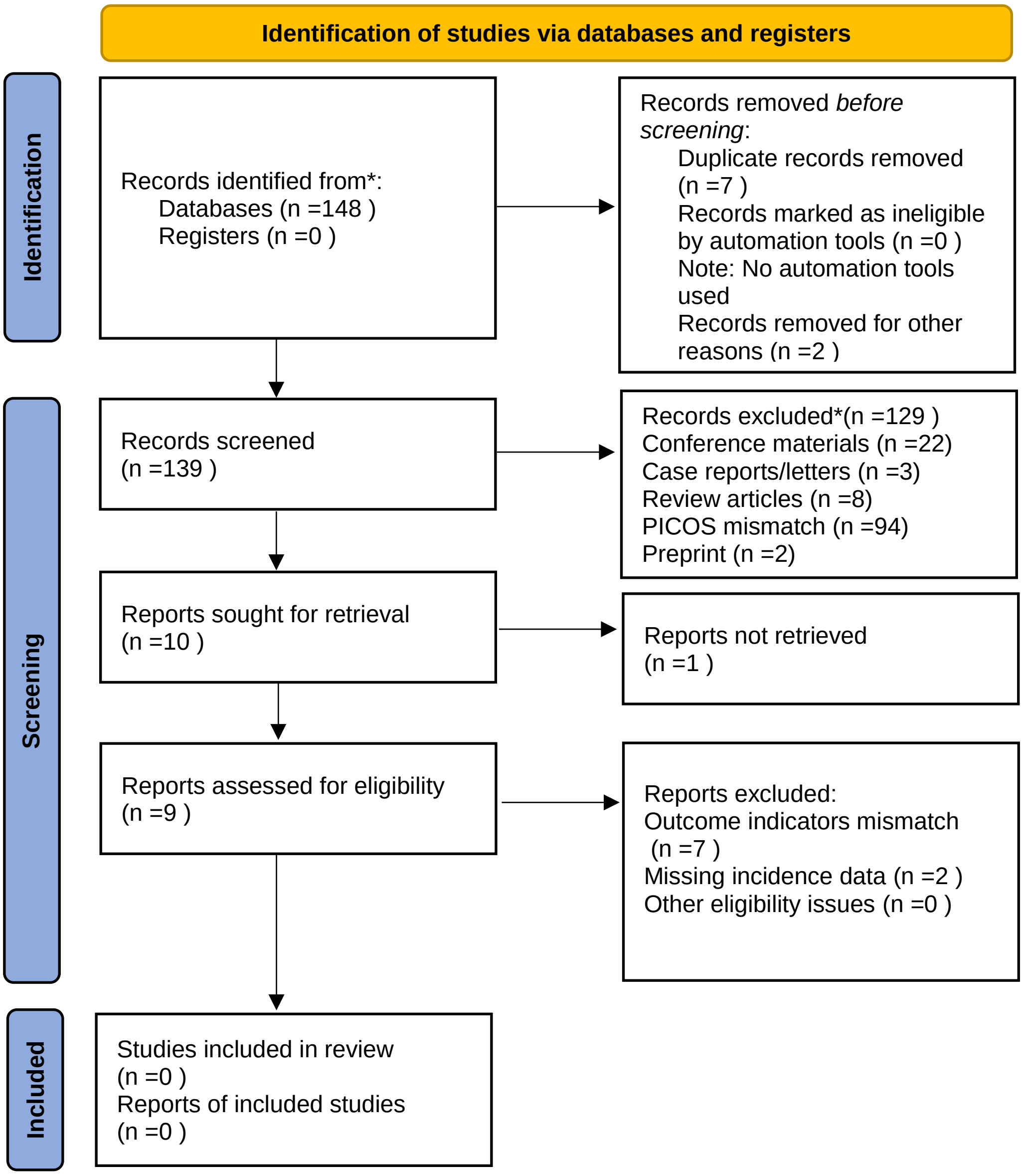

*Consider, if feasible to do so, reporting the number of records identified from each database or register searched (rather than the total number across all databases/registers).

**If automation tools were used, indicate how many records were excluded by a human and how many were excluded by automation tools.

Source: Page MJ, et al. BMJ 2021;372:n71. doi: 10.1136/bmj.n71.

This work is licensed under CC BY 4.0. To view a copy of this license, visit https://creativecommons.org/licenses/by/4.0/
