# Supplementary material for: Systematic review and meta-analysis of cardiovascular event incidence and risk factors in pediatric dialysis patients
Source: Medicine (Baltimore). 2025 Sep 19;104(38):e44545. doi: 10.1097/MD.0000000000044545 (PMC12459514; doi:10.1097/MD.0000000000044545)
Supplement: Supplementary file 2 [file medi-104-e44545-s002.pdf]

Create a new PROSPERO record...

Your PROSPERO registration records (1 record)

Registered 1 *What do these categories mean?*

Registered records published and stable

ID	Review	Date created	Last edited
CRD420251065118	Meta-analysis of the incidence and factors influencing cardiovascular-related events in pediatric and adolescent dialysis patients	1 June 2025	2 June 2025
