# Supplementary material for: Systematic review and meta-analysis of cardiovascular event incidence and risk factors in pediatric dialysis patients
Source: Medicine (Baltimore). 2025 Sep 19;104(38):e44545. doi: 10.1097/MD.0000000000044545 (PMC12459514; doi:10.1097/MD.0000000000044545)
Supplement: Supplementary file 3 [file medi-104-e44545-s003.docx]

Supplementary Material for: Meta-analysis of the incidence and factors influencing cardiovascular-related events in pediatric and adolescent dialysis patients

First Author: Xiaoying Zheng

Journal: Medicine

Pubmed retrieval strategy

| Search | Search terms |
| --- | --- |
| #1 | "Adolescent"[MeSH Terms] |
| #2 | "Adolescent"[MeSH Terms] OR "Adolescence"[Title/Abstract] OR "adolescent female"[Title/Abstract] OR "female adolescent"[Title/Abstract] OR "adolescent male"[Title/Abstract] OR "male adolescent"[Title/Abstract] OR "Youth"[Title/Abstract] OR "Teen"[Title/Abstract] OR "Teenager"[Title/Abstract] |
| #3 | "Renal Dialysis"[MeSH Terms] |
| #4 | "Renal Dialysis"[MeSH Terms] OR (("dialysance"[All Fields] OR "dialysances"[All Fields] OR "dialysation"[All Fields] OR "dialysator"[All Fields] OR "dialysators"[All Fields] OR "dialyse"[All Fields] OR "dialysed"[All Fields] OR "dialyser"[All Fields] OR "dialysers"[All Fields] OR "dialysing"[All Fields] OR "dialysis solutions"[Pharmacological Action] OR "dialysis solutions"[MeSH Terms] OR ("Dialysis"[All Fields] AND "solutions"[All Fields]) OR "dialysis solutions"[All Fields] OR "dialysate"[All Fields] OR "dialysates"[All Fields] OR "dialyzate"[All Fields] OR "dialyzates"[All Fields] OR "Dialysis"[MeSH Terms] OR "Dialysis"[All Fields] OR "Dialyses"[All Fields] OR "dialyzability"[All Fields] OR "dialyzable"[All Fields] OR "dialyzation"[All Fields] OR "dialyze"[All Fields] OR "dialyzed"[All Fields] OR "dialyzer"[All Fields] OR "dialyzer s"[All Fields] OR "dialyzers"[All Fields] OR "dialyzing"[All Fields] OR "Renal Dialysis"[MeSH Terms] OR ("Renal"[All Fields] AND "Dialysis"[All Fields]) OR "Renal Dialysis"[All Fields]) AND "Renal"[Title/Abstract]) OR "renal dialyses"[Title/Abstract] OR "dialysis renal"[Title/Abstract] OR "Hemodialysis"[Title/Abstract] OR "Hemodialyses"[Title/Abstract] OR "dialysis extracorporeal"[Title/Abstract] OR (("dialysance"[All Fields] OR "dialysances"[All Fields] OR "dialysation"[All Fields] OR "dialysator"[All Fields] OR "dialysators"[All Fields] OR "dialyse"[All Fields] OR "dialysed"[All Fields] OR "dialyser"[All Fields] OR "dialysers"[All Fields] OR "dialysing"[All Fields] OR "dialysis solutions"[Pharmacological Action] OR "dialysis solutions"[MeSH Terms] OR ("Dialysis"[All Fields] AND "solutions"[All Fields]) OR "dialysis solutions"[All Fields] OR "dialysate"[All Fields] OR "dialysates"[All Fields] OR "dialyzate"[All Fields] OR "dialyzates"[All Fields] OR "Dialysis"[MeSH Terms] OR "Dialysis"[All Fields] OR "Dialyses"[All Fields] OR "dialyzability"[All Fields] OR "dialyzable"[All Fields] OR "dialyzation"[All Fields] OR "dialyze"[All Fields] OR "dialyzed"[All Fields] OR "dialyzer"[All Fields] OR "dialyzer s"[All Fields] OR "dialyzers"[All Fields] OR "dialyzing"[All Fields] OR "Renal Dialysis"[MeSH Terms] OR ("Renal"[All Fields] AND "Dialysis"[All Fields]) OR "Renal Dialysis"[All Fields]) AND "Extracorporeal"[Title/Abstract]) OR "extracorporeal dialyses"[Title/Abstract] OR "extracorporeal dialysis"[Title/Abstract] |
| #5 | "Cardiovascular Diseases"[MeSH Terms] |
| #6 | "Cardiovascular Diseases"[MeSH Terms] OR "cardiovascular disease"[Title/Abstract] OR "disease cardiovascular"[Title/Abstract] OR "cardiac event"[Title/Abstract] OR "adverse cardiac event"[Title/Abstract] OR (("cardiacs"[All Fields] OR "heart"[MeSH Terms] OR "heart"[All Fields] OR "Cardiac"[All Fields]) AND "event adverse"[Title/Abstract]) OR "major adverse cardiac events"[Title/Abstract] |
| #7 | "Cross-Sectional Studies"[MeSH Terms] |
| #8 | "Cross-Sectional Studies"[MeSH Terms] OR "cross sectional study"[Title/Abstract] OR "studies cross sectional"[Title/Abstract] OR "cross sectional survey"[Title/Abstract] OR "survey cross sectional"[Title/Abstract] OR (("survey s"[All Fields] OR "surveyed"[All Fields] OR "surveying"[All Fields] OR "surveys and questionnaires"[MeSH Terms] OR ("surveys"[All Fields] AND "questionnaires"[All Fields]) OR "surveys and questionnaires"[All Fields] OR "Survey"[All Fields] OR "surveys"[All Fields]) AND "disease frequency"[Title/Abstract]) OR "disease frequency survey"[Title/Abstract] OR "analysis cross sectional"[Title/Abstract] OR "analyses cross sectional"[Title/Abstract] OR "cross sectional analysis"[Title/Abstract] OR "cross sectional analyses"[Title/Abstract] OR "prevalence study"[Title/Abstract] OR "studies prevalence"[Title/Abstract] |
| #9 | "Cohort Studies"[MeSH Terms] |
| #10 | "Cohort Studies"[MeSH Terms] OR "cohort study"[Title/Abstract] OR "studies cohort"[Title/Abstract] OR "concurrent study"[Title/Abstract] OR "concurrent studies"[Title/Abstract] OR "closed cohort studies"[Title/Abstract] OR (("Cohort"[All Fields] OR "cohort s"[All Fields] OR "cohorte"[All Fields] OR "cohorts"[All Fields]) AND "study closed"[Title/Abstract]) OR "historical cohort studies"[Title/Abstract] OR (("Cohort"[All Fields] OR "cohort s"[All Fields] OR "cohorte"[All Fields] OR "cohorts"[All Fields]) AND "studies historical"[Title/Abstract]) OR "study historical cohort"[Title/Abstract] OR "incidence studies"[Title/Abstract] OR "incidence study"[Title/Abstract] OR "study incidence"[Title/Abstract] OR "analysis cohort"[Title/Abstract] OR "cohort analyses"[Title/Abstract] OR "birth cohort studies"[Title/Abstract] OR (("Cohort"[All Fields] OR "cohort s"[All Fields] OR "cohorte"[All Fields] OR "cohorts"[All Fields]) AND "studies birth"[Title/Abstract]) OR "study birth cohort"[Title/Abstract] OR "study birth cohort"[Title/Abstract] |
| #11 | "Cohort Studies"[MeSH Terms] OR "cohort study"[Title/Abstract] OR "studies cohort"[Title/Abstract] OR "concurrent study"[Title/Abstract] OR "concurrent studies"[Title/Abstract] OR "closed cohort studies"[Title/Abstract] OR (("Cohort"[All Fields] OR "cohort s"[All Fields] OR "cohorte"[All Fields] OR "cohorts"[All Fields]) AND "study closed"[Title/Abstract]) OR "historical cohort studies"[Title/Abstract] OR (("Cohort"[All Fields] OR "cohort s"[All Fields] OR "cohorte"[All Fields] OR "cohorts"[All Fields]) AND "studies historical"[Title/Abstract]) OR "study historical cohort"[Title/Abstract] OR "incidence studies"[Title/Abstract] OR "incidence study"[Title/Abstract] OR "study incidence"[Title/Abstract] OR "analysis cohort"[Title/Abstract] OR "cohort analyses"[Title/Abstract] OR "birth cohort studies"[Title/Abstract] OR (("Cohort"[All Fields] OR "cohort s"[All Fields] OR "cohorte"[All Fields] OR "cohorts"[All Fields]) AND "studies birth"[Title/Abstract]) OR "study birth cohort"[Title/Abstract] OR "study birth cohort"[Title/Abstract] OR ("Cross-Sectional Studies"[MeSH Terms] OR "cross sectional study"[Title/Abstract] OR "studies cross sectional"[Title/Abstract] OR "cross sectional survey"[Title/Abstract] OR "survey cross sectional"[Title/Abstract] OR (("survey s"[All Fields] OR "surveyed"[All Fields] OR "surveying"[All Fields] OR "surveys and questionnaires"[MeSH Terms] OR ("surveys"[All Fields] AND "questionnaires"[All Fields]) OR "surveys and questionnaires"[All Fields] OR "Survey"[All Fields] OR "surveys"[All Fields]) AND "disease frequency"[Title/Abstract]) OR "disease frequency survey"[Title/Abstract] OR "analysis cross sectional"[Title/Abstract] OR "analyses cross sectional"[Title/Abstract] OR "cross sectional analysis"[Title/Abstract] OR "cross sectional analyses"[Title/Abstract] OR "prevalence study"[Title/Abstract] OR "studies prevalence"[Title/Abstract]) |
| #12 | "Child"[MeSH Terms] |
| #13 | "Child"[MeSH Terms] OR "children"[Title/Abstract] |
| #14 | "Child"[MeSH Terms] OR "children"[Title/Abstract] OR "Adolescent"[MeSH Terms] OR "Adolescence"[Title/Abstract] OR "adolescent female"[Title/Abstract] OR "female adolescent"[Title/Abstract] OR "adolescent male"[Title/Abstract] OR "male adolescent"[Title/Abstract] OR "Youth"[Title/Abstract] OR "Teen"[Title/Abstract] OR "Teenager"[Title/Abstract] |
| #15 | ("Renal Dialysis"[MeSH Terms] OR (("dialysance"[All Fields] OR "dialysances"[All Fields] OR "dialysation"[All Fields] OR "dialysator"[All Fields] OR "dialysators"[All Fields] OR "dialyse"[All Fields] OR "dialysed"[All Fields] OR "dialyser"[All Fields] OR "dialysers"[All Fields] OR "dialysing"[All Fields] OR "dialysis solutions"[Pharmacological Action] OR "dialysis solutions"[MeSH Terms] OR ("Dialysis"[All Fields] AND "solutions"[All Fields]) OR "dialysis solutions"[All Fields] OR "dialysate"[All Fields] OR "dialysates"[All Fields] OR "dialyzate"[All Fields] OR "dialyzates"[All Fields] OR "Dialysis"[MeSH Terms] OR "Dialysis"[All Fields] OR "Dialyses"[All Fields] OR "dialyzability"[All Fields] OR "dialyzable"[All Fields] OR "dialyzation"[All Fields] OR "dialyze"[All Fields] OR "dialyzed"[All Fields] OR "dialyzer"[All Fields] OR "dialyzer s"[All Fields] OR "dialyzers"[All Fields] OR "dialyzing"[All Fields] OR "Renal Dialysis"[MeSH Terms] OR ("Renal"[All Fields] AND "Dialysis"[All Fields]) OR "Renal Dialysis"[All Fields]) AND "Renal"[Title/Abstract]) OR "renal dialyses"[Title/Abstract] OR "dialysis renal"[Title/Abstract] OR "Hemodialysis"[Title/Abstract] OR "Hemodialyses"[Title/Abstract] OR "dialysis extracorporeal"[Title/Abstract] OR (("dialysance"[All Fields] OR "dialysances"[All Fields] OR "dialysation"[All Fields] OR "dialysator"[All Fields] OR "dialysators"[All Fields] OR "dialyse"[All Fields] OR "dialysed"[All Fields] OR "dialyser"[All Fields] OR "dialysers"[All Fields] OR "dialysing"[All Fields] OR "dialysis solutions"[Pharmacological Action] OR "dialysis solutions"[MeSH Terms] OR ("Dialysis"[All Fields] AND "solutions"[All Fields]) OR "dialysis solutions"[All Fields] OR "dialysate"[All Fields] OR "dialysates"[All Fields] OR "dialyzate"[All Fields] OR "dialyzates"[All Fields] OR "Dialysis"[MeSH Terms] OR "Dialysis"[All Fields] OR "Dialyses"[All Fields] OR "dialyzability"[All Fields] OR "dialyzable"[All Fields] OR "dialyzation"[All Fields] OR "dialyze"[All Fields] OR "dialyzed"[All Fields] OR "dialyzer"[All Fields] OR "dialyzer s"[All Fields] OR "dialyzers"[All Fields] OR "dialyzing"[All Fields] OR "Renal Dialysis"[MeSH Terms] OR ("Renal"[All Fields] AND "Dialysis"[All Fields]) OR "Renal Dialysis"[All Fields]) AND "Extracorporeal"[Title/Abstract]) OR "extracorporeal dialyses"[Title/Abstract] OR "extracorporeal dialysis"[Title/Abstract]) AND ("Cardiovascular Diseases"[MeSH Terms] OR "cardiovascular disease"[Title/Abstract] OR "disease cardiovascular"[Title/Abstract] OR "cardiac event"[Title/Abstract] OR "adverse cardiac event"[Title/Abstract] OR (("cardiacs"[All Fields] OR "heart"[MeSH Terms] OR "heart"[All Fields] OR "Cardiac"[All Fields]) AND "event adverse"[Title/Abstract]) OR "major adverse cardiac events"[Title/Abstract]) AND ("Cohort Studies"[MeSH Terms] OR "cohort study"[Title/Abstract] OR "studies cohort"[Title/Abstract] OR "concurrent study"[Title/Abstract] OR "concurrent studies"[Title/Abstract] OR "closed cohort studies"[Title/Abstract] OR (("Cohort"[All Fields] OR "cohort s"[All Fields] OR "cohorte"[All Fields] OR "cohorts"[All Fields]) AND "study closed"[Title/Abstract]) OR "historical cohort studies"[Title/Abstract] OR (("Cohort"[All Fields] OR "cohort s"[All Fields] OR "cohorte"[All Fields] OR "cohorts"[All Fields]) AND "studies historical"[Title/Abstract]) OR "study historical cohort"[Title/Abstract] OR "incidence studies"[Title/Abstract] OR "incidence study"[Title/Abstract] OR "study incidence"[Title/Abstract] OR "analysis cohort"[Title/Abstract] OR "cohort analyses"[Title/Abstract] OR "birth cohort studies"[Title/Abstract] OR (("Cohort"[All Fields] OR "cohort s"[All Fields] OR "cohorte"[All Fields] OR "cohorts"[All Fields]) AND "studies birth"[Title/Abstract]) OR "study birth cohort"[Title/Abstract] OR "study birth cohort"[Title/Abstract] OR ("Cross-Sectional Studies"[MeSH Terms] OR "cross sectional study"[Title/Abstract] OR "studies cross sectional"[Title/Abstract] OR "cross sectional survey"[Title/Abstract] OR "survey cross sectional"[Title/Abstract] OR (("survey s"[All Fields] OR "surveyed"[All Fields] OR "surveying"[All Fields] OR "surveys and questionnaires"[MeSH Terms] OR ("surveys"[All Fields] AND "questionnaires"[All Fields]) OR "surveys and questionnaires"[All Fields] OR "Survey"[All Fields] OR "surveys"[All Fields]) AND "disease frequency"[Title/Abstract]) OR "disease frequency survey"[Title/Abstract] OR "analysis cross sectional"[Title/Abstract] OR "analyses cross sectional"[Title/Abstract] OR "cross sectional analysis"[Title/Abstract] OR "cross sectional analyses"[Title/Abstract] OR "prevalence study"[Title/Abstract] OR "studies prevalence"[Title/Abstract])) AND ("Child"[MeSH Terms] OR "children"[Title/Abstract] OR ("Adolescent"[MeSH Terms] OR "Adolescence"[Title/Abstract] OR "adolescent female"[Title/Abstract] OR "female adolescent"[Title/Abstract] OR "adolescent male"[Title/Abstract] OR "male adolescent"[Title/Abstract] OR "Youth"[Title/Abstract] OR "Teen"[Title/Abstract] OR "Teenager"[Title/Abstract])) |

Embase retrieval strategy

| Search | Search terms |
| --- | --- |
| #1 | 'child'/exp |
| #2 | child:ab,ti OR children:ab,ti |
| #3 | 'adolescent'/exp |
| #4 | adolescent:ab,ti OR teenager:ab,ti |
| #5 | #3 OR #4 |
| #6 | #1 OR #2 |
| #7 | #5 OR #6 |
| #8 | 'hemodialysis'/exp |
| #9 | hemodialysis:ab,ti OR 'blood dialysis':ab,ti OR 'dialysis center':ab,ti OR 'extracorporeal blood cleansing':ab,ti OR 'extracorporeal dialysis':ab,ti OR haemodialysis:ab,ti OR hemodialyse:ab,ti OR hemorenodialysis:ab,ti OR hemotrialysate:ab,ti OR 'renal dialysis':ab,ti |
| #10 | #8 OR #9 |
| #11 | 'cardiovascular disease'/exp |
| #12 | 'cardiovascular disease':ab,ti OR angiocardiopathy:ab,ti OR 'angiocardiovascular disease':ab,ti OR 'cardiovascular complication':ab,ti OR 'cardiovascular disorder':ab,ti OR 'cardiovascular disturbance':ab,ti OR 'cardiovascular lesion':ab,ti OR 'cardiovascular syndrome':ab,ti OR 'cardiovascular vegetative disorder':ab,ti OR 'major adverse cardiovascular event':ab,ti OR 'complication, cardiovascular':ab,ti OR 'disease, cardiovascular':ab,ti |
| #13 | #11 OR #12 |
| #14 | 'cross-sectional study'/exp |
| #15 | 'cross-sectional design':ab,ti OR 'cross-sectional study':ab,ti OR 'cross-sectional research':ab,ti OR 'cross-sectional studies':ab,ti |
| #16 | #14 OR #15 |
| #17 | 'cohort analysis'/exp |
| #18 | 'cohort analysis':ab,ti OR 'analysis, cohort':ab,ti OR 'cohort fertility':ab,ti OR 'cohort life cycle':ab,ti OR 'cohort studies':ab,ti OR 'cohort study':ab,ti OR 'fertility, cohort':ab,ti |
| #19 | #17 OR #18 |
| #20 | #16 OR #19 |
| #21 | #7 AND #10 AND #13 AND #20 |

Web of science retrieval strategy

| Search | Search terms |
| --- | --- |
| #1 | child (Topic) OR Children (Topic) OR Adolescent (Topic) OR Adolescence (Topic) OR adolescent female (Topic) OR female adolescent (Topic) OR adolescent male (Topic) OR male adolescent (Topic) OR Youth (Topic) OR Teen (Topic) OR Teenager (Topic) and Preprint Citation Index (Exclude – Database) |
| #2 | Renal Dialysis (Topic) OR Renal Dialyses (Topic) OR Hemodialysis (Topic) OR Hemodialyses (Topic) OR Extracorporeal Dialyses (Topic) OR Extracorporeal Dialysis (Topic) and Preprint Citation Index (Exclude – Database) |
| #3 | Cardiovascular Diseases (Topic) OR cardiovascular disease (Topic) OR cardiac event (Topic) OR adverse cardiac event (Topic) and Preprint Citation Index (Exclude – Database) |
| #4 | Cross-Sectional Studies (Topic) OR Cross Sectional Studies (Topic) OR Cross-Sectional Study (Topic) OR Cross-Sectional Survey (Topic) OR Cross Sectional Survey (Topic) OR Disease Frequency Survey (Topic) OR Cross-Sectional Analyses (Topic) OR Cross Sectional Analysis (Topic) OR Cross Sectional Analyses (Topic) OR Prevalence Studies (Topic) OR Prevalence Study (Topic) OR cohort studies (Topic) OR Cohort Study (Topic) OR Concurrent Study (Topic) OR Concurrent Studies (Topic) OR Closed Cohort Studies (Topic) OR Closed Cohort Study (Topic) OR Historical Cohort Studies (Topic) OR Historical Cohort Study (Topic) OR Incidence Studies (Topic) OR Cohort Analyses (Topic) OR Birth Cohort Studies (Topic) OR Birth Cohort Study (Topic) OR Cohort Analysis (Topic) and Preprint Citation Index (Exclude – Database) |
| #5 | #1 AND #2 AND #3 AND #4 and Preprint Citation Index (Exclude – Database) |

Cochrane retrieval strategy

| Search | Search terms |
| --- | --- |
| #1 | MeSH descriptor: [Child] explode all trees |
| #2 | (child):ti,ab,kw OR (children):ti,ab,kw |
| #3 | .MeSH descriptor: [Adolescent] explode all trees |
| #4 | .(Adolescent):ti,ab,kw OR (Male Adolescent):ti,ab,kw OR (Adolescents):ti,ab,kw OR (Male Adolescent):ti,ab,kw OR (Adolescents, Male):ti,ab,kw |
| #5 | (Adolescent, Male):ti,ab,kw OR (Adolescence):ti,ab,kw OR (Teen):ti,ab,kw OR (Teenager):ti,ab,kw OR (Teenagers):ti,ab,kw |
| #6 | (Female Adolescents):ti,ab,kw OR (Adolescent, Female):ti,ab,kw OR (Female Adolescent):ti,ab,kw OR (Adolescents, Female):ti,ab,kw OR (Youth):ti,ab,kw |
| #7 | #3 or #4 or #5 or #6 |
| #8 | #1 or #2 |
| #9 | #7 or #8 |
| #10 | MeSH descriptor: [Renal Dialysis] explode all trees |
| #11 | (Renal Dialysis):ti,ab,kw OR (Hemodialyses):ti,ab,kw OR (Hemodialysis):ti,ab,kw OR (Extracorporeal Dialyses):ti,ab,kw OR (Extracorporeal Dialysis):ti,ab,kw |
| #12 | (Dialysis, Extracorporeal):ti,ab,kw OR (Dialyses, Extracorporeal):ti,ab,kw OR (Renal Dialyses):ti,ab,kw OR (Dialysis, Renal):ti,ab,kw OR (Dialyses, Renal):ti,ab,kw |
| #13 | #10 or #11 or #12 |
| #14 | MeSH descriptor: [Cardiovascular Diseases] explode all trees |
| #15 | (Cardiovascular Diseases):ti,ab,kw OR (Major Adverse Cardiac Events):ti,ab,kw OR (Adverse Cardiac Event):ti,ab,kw OR (Cardiac Event, Adverse):ti,ab,kw OR (Cardiac Events, Adverse):ti,ab,kw |
| #16 | (Event, Cardiac):ti,ab,kw OR (Cardiac Events):ti,ab,kw OR (Cardiac Event):ti,ab,kw OR (Cardiovascular Disease):ti,ab,kw OR (Disease, Cardiovascular):ti,ab,kw |
| #17 | #14 or #15 or #16 |
| #18 | MeSH descriptor: [Cross-Sectional Studies] explode all trees |
| #19 | (Cross-Sectional Studies):ti,ab,kw OR (Prevalence Study):ti,ab,kw OR (Study, Prevalence):ti,ab,kw OR (Prevalence Studies):ti,ab,kw OR (Analysis, Cross Sectional):ti,ab,kw |
| #20 | (Disease Frequency Survey):ti,ab,kw OR (Analysis, Cross-Sectional):ti,ab,kw OR (Surveys, Cross-Sectional):ti,ab,kw OR (Analyses, Cross Sectional):ti,ab,kw OR (Study, Cross-Sectional):ti,ab,kw |
| #21 | (Cross Sectional Survey):ti,ab,kw OR (Cross-Sectional Analysis):ti,ab,kw OR (Surveys, Disease Frequency):ti,ab,kw OR (Cross-Sectional Study):ti,ab,kw OR (Survey, Cross-Sectional):ti,ab,kw |
| #22 | (Studies, Cross-Sectional):ti,ab,kw OR (Survey, Disease Frequency):ti,ab,kw OR (Cross Sectional Analyses):ti,ab,kw OR (Cross Sectional Studies):ti,ab,kw OR (Disease Frequency Surveys):ti,ab,kw |
| #23 | (Analyses, Cross-Sectional):ti,ab,kw OR (Cross Sectional Analysis):ti,ab,kw OR (Cross-Sectional Survey):ti,ab,kw OR (Cross-Sectional Analyses):ti,ab,kw OR (Cross-Sectional Surveys):ti,ab,kw |
| #25 | MeSH descriptor: [Cohort Studies] explode all trees |
| #26 | (cohort studies):ti,ab,kw OR (Cohort Studies, Birth):ti,ab,kw OR (Birth Cohort Studies):ti,ab,kw OR (Study, Birth Cohort):ti,ab,kw OR (Cohort Study, Closed):ti,ab,kw |
| #27 | (Closed Cohort Studies):ti,ab,kw OR (Closed Cohort Study):ti,ab,kw OR (Cohort Studies, Closed):ti,ab,kw OR (Study, Closed Cohort):ti,ab,kw OR (Studies, Closed Cohort):ti,ab,kw |
| #28 | (Cohort Analyses):ti,ab,kw OR (Cohort Analysis):ti,ab,kw OR (Analysis, Cohort):ti,ab,kw OR (Analyses, Cohort):ti,ab,kw OR (Study, Historical Cohort):ti,ab,kw |
| #29 | (Historical Cohort Study):ti,ab,kw OR (Cohort Study, Historical):ti,ab,kw OR (Studies, Historical Cohort):ti,ab,kw OR (Cohort Studies, Historical):ti,ab,kw OR (Historical Cohort Studies):ti,ab,kw |
| #30 | .(Concurrent Study):ti,ab,kw OR (Concurrent Studies):ti,ab,kw OR (Studies, Concurrent):ti,ab,kw OR (Cohort Study):ti,ab,kw OR (Studies, Cohort):ti,ab,kw |
| #31 | (Study, Concurrent):ti,ab,kw OR (Study, Cohort):ti,ab,kw OR (Incidence Study):ti,ab,kw OR (Study, Incidence):ti,ab,kw |
| #32 | #25 or #26 or #27 or #28 or #29 or #30 or #31 |
| #33 | #24 or #32 |
| #34 | #9 and #13 and #17 and #33 |
