# Supplementary material for: Systematic review and meta-analysis of cardiovascular event incidence and risk factors in pediatric dialysis patients
Source: Medicine (Baltimore). 2025 Sep 19;104(38):e44545. doi: 10.1097/MD.0000000000044545 (PMC12459514; doi:10.1097/MD.0000000000044545)
Supplement: Supplementary file 4 [file medi-104-e44545-s004.docx]

**Table S1: Results of the quality assessment of the literature included in the cross-sectional study.**

**The scale consists of 11 questions, each of which can be answered“yes”or“no”or“not sure”on a scale of 0-11.**

| **Study** | **Responsive** | | | | | | | | | | | |
| --- | --- | --- | --- | --- | --- | --- | --- | --- | --- | --- | --- | --- |
|  | **Q1** | **Q2** | **Q3** | **Q4** | **Q5** | **Q6** | **Q7** | **Q8** | **Q9** | **Q10** | **Q11** | **得分** |
| Johnstone（1996） | Y | Y | Y | N | U | Y | Y | Y | Y | Y | U | 8 |
| Mitsnefes(2000) | Y | Y | Y | N | U | N | N | U | Y | Y | U | 5 |
| Katsoufis (2014) | Y | Y | Y | N | U | Y | N | Y | Y | Y | U | 8 |
| Badawy（2020） | Y | Y | Y | N | U | N | N | U | Y | Y | U | 5 |
| Bakkaloglu (2011) | Y | N | Y | N | U | Y | N | Y | Y | Y | Y | 7 |

Note: Q1-Q11 represent the questions used to assess the quality of the cross-sectional study according to the AHRQ scale as listed below:

Q1:Is the source of information (survey, literature review) specified?

Q2:Are inclusion and exclusion criteria for exposed and non-exposed groups (cases and controls) listed or referenced in previous publications?

Q3:Is the time period for identifying patients given (which time period patients were included in the study)?

Q4:Is the study population continuous (were all patients within a certain time period included in the study) if not a population source?

Q5:Was the assessor of the patient's subjectivized indicators isolated from the patient's other objective indicators (e.g.when assessing pain levels the assessor could not be informed of the patient's fracture site radiograph results)?

Q6:Describe any assessments performed for quality assurance purposes (e.g. testing/retesting of primary outcome indicators)?

Q7:Did the study estimate the sample size or provide a rationale for it?

Q8:Does it describe the validity and reliability of the exposure or outcome measures?

Q9:Were statistical methods used to adjust for the effects of confounders?

Q10:Were all significant outcome indicators reported?

Q11:Summarized patient response rates and completeness of data collection?

Y :yes; N: no; U :unclear;

**Table S2: Results of the quality assessment of the literature of the included cohort studies.**

**Each study received a maximum of one“*”per entry for“selection”and“outcome”and a maximum of two“*”for“comparability”. A maximum of two “*”were allowed for each entry for“Comparability”. Quality assessment scores ranged from 0-9.**

| Study | Option | | | | Comparability | Conclusion | | | Score |
| --- | --- | --- | --- | --- | --- | --- | --- | --- | --- |
|  | (1)Representation of the Exposure Queue | (2)Selection of non-exposed queues | (3)Determination of exposure factors | (4)No outcome disease at study entry | (1)Adjustment for confounders based on design or analysis | (1)Assessment of the outcome | (2)Whether the follow-up period is long enough | (3)Completeness of follow-up |  |
| Chavers（2002） | * | 0 | * | 0 | * | * | * | 0 | 5 |
| Galiyeva（2019） | * | 0 | * | * | * | * | * | * | 7 |
| Li（2023） | * | 0 | * | * | * | * | * | 0 | 6 |
| Chesnaye（2016） | * | 0 | * | * | ** | * | * | * | 8 |
| Seeherunvong (2012) | * | 0 | * | 0 | ** | * | 0 | 0 | 5 |
